# Supplementary material for: Metabolome and Transcriptome Profiling Reveal Carbon Metabolic Flux Changes in Yarrowia lipolytica Cells to Rapamycin
Source: J Fungi (Basel). 2022 Sep 6;8(9):939. doi: 10.3390/jof8090939 (PMC9504542; doi:10.3390/jof8090939)
Supplement: Supplementary file 1 [file jof-08-00939-s001.zip › Table S1.pdf]

Table S1: The primers used in this study.

Name	Sequence
YALI0_B04202g-F	CAACATTCTGGTGACTCTGA
YALI0_B04202g-R	TAGCGTAGGTGGTGAAGG
YALI0_B05654g-F	TCTTCGGTATCGGTGTGA
YALI0_B05654g-R	GCCATCCTCCAGAGTAGT
YALI0_B08899g-F	GCCTACCAGTCCTTCTACA
YALI0_B08899g-R	CCAGAATCAGCGTGTTGA
YALI0_B10494g-F	CCTCATACGAAGAACATTGTG
YALI0_B10494g-R	CGGAGCAGGTAGTGAAGT
YALI0_B20328g-F	ACTCAGAGCCAGTCAACT
YALI0_B20328g-R	GTGTGCCAGTAGGTCTTC
YALI0_B21846g-F	TTCTCTGCTGCTGTCAAG
YALI0_B21846g-R	CGTTATCCATCTCGTTCCA
YALI0_C13706g-F	CCAAGAACATCAAGAAGAAGG
YALI0_C13706g-R	CAGAGTGTAGTCCGAGG
YALI0_D07326g-F	CAAGGAGATTCACGAGGTT
YALI0_D07326g-R	CAGACACAGCACAGACAA
YALI0_E33935g-F	CGTCGTGGTCATCTATCG
YALI0_E33935g-R	GGTTGGAGTCGGTCATTG
YALI0_F19866g-F	ATCAAGACCACCTCCAAGT
YALI0_F19866g-R	GAAGAGAAGAGAACCGACAA
YALI0_F24255g-F	TTGGTGACGACTGTGGTA
YALI0_F24255g-R	GAAGAAGCAGCAGAAGAGG
26S 5	GGGAAGGAAATGAGTGGAGAGTGG
26S 3	GTGGATTATGTCGTCGGTGGCA
