# Supplementary material for: Metabolome and Transcriptome Profiling Reveal Carbon Metabolic Flux Changes in Yarrowia lipolytica Cells to Rapamycin
Source: J Fungi (Basel). 2022 Sep 6;8(9):939. doi: 10.3390/jof8090939 (PMC9504542; doi:10.3390/jof8090939)
Supplement: Supplementary file 1 [file jof-08-00939-s001.zip › Table S8.pdf]

Table S8: Enzymes and genes related to amino acid and lipids metabolism.

Enzymes	Genes	Annotation
HK1	*YALI0_B22308g*	Hexokinase
HK2	*YALI0_E20207g*	Hexokinase
HK3	*YALI0_E15488g*	Hexokinase
ACL1	*YALI0_E34793g*	ATP citrate lyase subunit 1
ACL2	*YALI0_D24431g*	ATP citrate lyase subunit 2
FUM	*YALI0_C06776g*	Fumarate hydratase
SDH	*YALI0_D23397g*	Succinate dehydrogenase
PCK	*YALI0_C16995g*	phosphoenolpyruvate carboxykinase (ATP)
PRPS	*YALI0_B00836g*	ribose-phosphate pyrophosphokinase
HISG	*YALI0_C05170g*	ATP phosphoribosyltransferase
AROF	*YALI0_B20020g*	3-deoxy-7-phosphoheptulonate synthase
ARO8	*YALI0_E20977g*	aromatic amino acid aminotransferase I/2-aminoadipate transaminase
ARO9	*YALI0_C05258g*	aromatic amino acid aminotransferase II
TRP3	*YALI0_E14751g*	anthranilate synthase / indole-3-glycerol phosphate synthase
GOT1	*YALI0_F29337g*	aspartate aminotransferase, cytoplasmic
ILVE	*YALI0_D01265g*	branched-chain amino acid aminotransferase
ILVD	*YALI0_C23408g*	dihydroxy-acid dehydratase
ILVB	*YALI0_C00253g*	acetolactate synthase I/II/III large subunit
ILVC	*YALI0_D03135g*	ketol-acid reductoisomerase
ALT	*YALI0_D06325g*	alanine transaminase
GDHA	*YALI0_F17820g*	glutamate dehydrogenase (NADP+)
GUDB	*YALI0_E09603g*	glutamate dehydrogenase
GLNA1	*YALI0_D13024g*	glutamine synthetase
GLNA2	*YALI0_F00506g*	glutamine synthetase
ARG1	*YALI0_E07535g*	arginase
OTC	*YALI0_D14894g*	ornithine carbamoyltransferase
ACC	*YALI0_C11407g*	acetyl-CoA carboxylase
FABF	*YALI0_F30679g*	3-oxoacyl-[acyl-carrier-protein] synthase II
FAS2	*YALI0_B19382g*	fatty acid synthase subunit alpha,
FAS1	*YALI0_B15059g*	fatty acid synthase subunit beta
ACOX3	*YALI0_C23859g*	acyl-CoA oxidase
ACOX1	*YALI0_D24750g*	acyl-CoA oxidase
ACOX2	*YALI0_F10857g*	acyl-CoA oxidase
ACOX4	*YALI0_E32835g*	acyl-CoA oxidase
ECHS1	*YALI0_B10406g*	enoyl-CoA hydratase
ACAA1	*YALI0_E18568g*	acetyl-CoA acyltransferase 1
ACAT1	*YALI0_E11099g*	acetyl-CoA C-acetyltransferase
ACAT2	*YALI0_B08536g*	acetyl-CoA C-acetyltransferase
DGAT1	*YALI0_D07986g*	diacylglycerol O-acyltransferase 1
ATG15	*YALI0_F06358g*	lipase ATG15
TGL3	*YALI0_D17534g*	TAG lipase

TGL2	*YALI0_E31515g*	triacylglycerol lipase
ERG25	*YALI0_F11297g*	methylsterol monooxygenase
ERG2a	*YALI0_E32065g*	C-8 sterol isomerase
ERG24	*YALI0_B23298g*	Delta14-sterol reductase
CYP51	*YALI0_B05126g*	sterol 14alpha-demethylase
SOAT	*YALI0_F06578g*	sterol O-acyltransferase
ERG2b	*YALI0_B17204g*	C-8 sterol isomerase
LIPA	*YALI0_E32035g*	lysosomal acid lipase/cholesteryl ester hydrolase
FAD2	*YALI0_B10153g*	omega-6 fatty acid desaturase
PROB	*YALI0_A19206g*	glutamate 5-kinase
PCDH	*YALI0_B09647g*	1-pyrroline-5-carboxylate dehydrogenase
PRODH	*YALI0_B09625g*	proline dehydrogenase
