# Supplementary material for: Metabolome and Transcriptome Profiling Reveal Carbon Metabolic Flux Changes in Yarrowia lipolytica Cells to Rapamycin
Source: J Fungi (Basel). 2022 Sep 6;8(9):939. doi: 10.3390/jof8090939 (PMC9504542; doi:10.3390/jof8090939)
Supplement: Supplementary file 1 [file jof-08-00939-s001.zip › Table S9.pdf]

Table S9: Abbreviations for metabolites.

Abbreviation	Annotation
G6P	Glucose 6-phosphate
GA3P	D-Glyceraldehyde 3-phosphate
αKG	alpha-Ketoglutaric acid
OAA	Oxaloacetic acid
6PG	6-Phospho-D-gluconate
E4P	Erythrose 4-phosphate
Shi	Shikimate;Shikimic acid
Cho	Chorismate
G3P	Glycerate 3-phosphate
ASH	L-Aspartate 4-semialdehyde
TH	L-2,3,4,5-Tetrahydrodipicolinate
DAP	LL-2,6-Diaminopimelate
DMP	2,3-Dihydroxy-3-methylpentanoate
AcGlu	N-Acetyl-L-glutamic acid
AcGSA	N-Acetyl-L-glutamate 5-semialdehyde
AcCit	N-Acetyl-L-citrulline
Cit	Citrulline
AS	L-Argininosuccinate;
Orn	L-Ornithine;
CA	Citric acid
PalACP	Palmitoyl-acyl-carrier protein
TAG	Triacylglycerol
FA	Fatty acid
PrePP	Presqualene diphosphate
SE	Squalene 2,3-epoxide
Zym	Zymosterol
Erg	Ergosterol
GSA	L-Glutamic gamma-semialdehyde
CE	Cholesterol ester
Cam	Campesterol
Chol	Cholesterol
