# Supplementary figures and images for: Metabolome and Transcriptome Profiling Reveal Carbon Metabolic Flux Changes in Yarrowia lipolytica Cells to Rapamycin
Source: J Fungi (Basel). 2022 Sep 6;8(9):939. doi: 10.3390/jof8090939 (PMC9504542; doi:10.3390/jof8090939)

Figure S1: PCA score plots and RSD of QC samples.

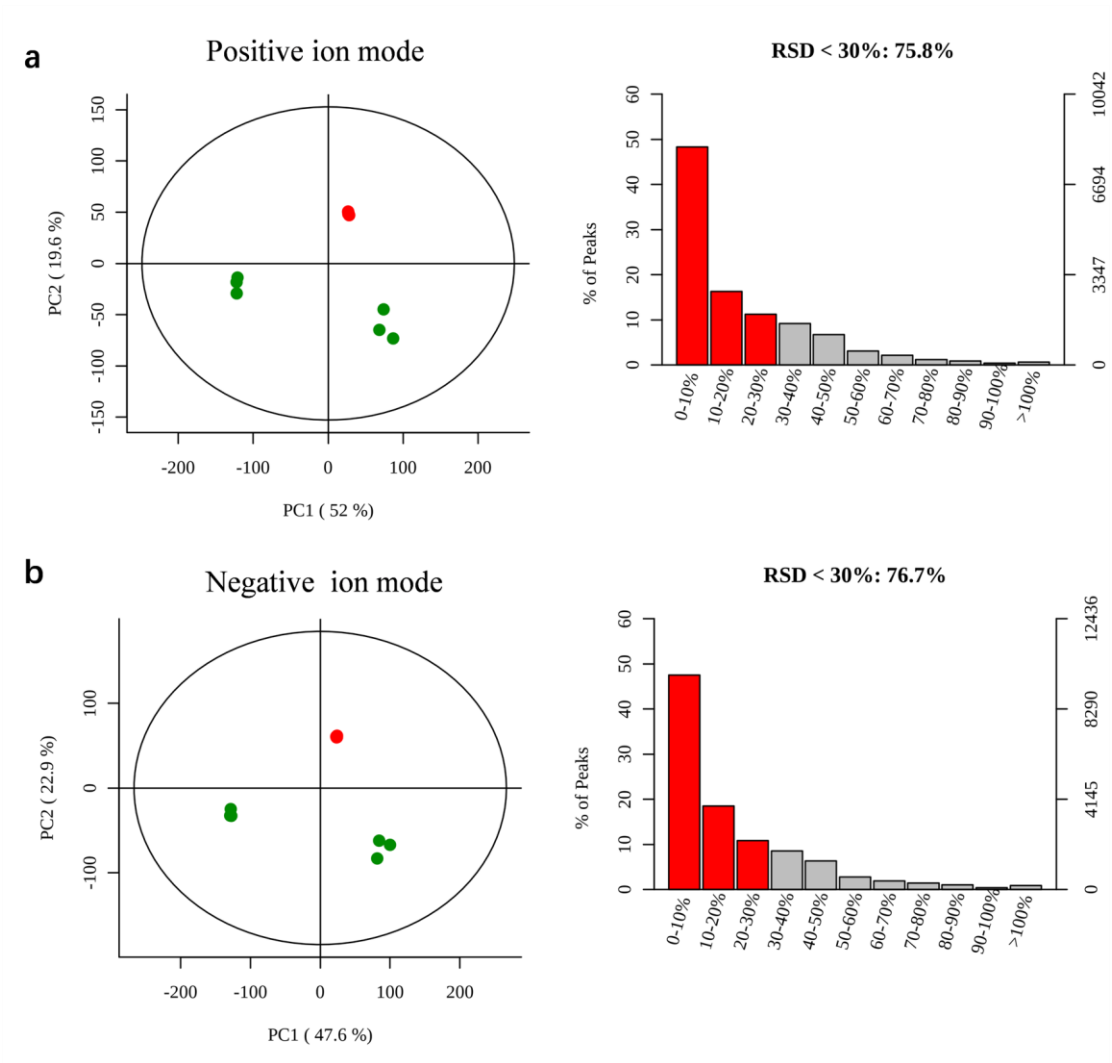

Supplement: Supplementary file 1 [file jof-08-00939-s001.zip › Figure S1.pdf]
